# Phlebotomine sand fly survey in the Republic of Moldova: species composition, distribution and host preferences

**DOI:** 10.1186/s13071-021-04858-4

**Published:** 2021-07-21

**Authors:** Tatiana Șuleșco, Ozge Erisoz Kasap, Petr Halada, Gizem Oğuz, Dimian Rusnac, Marketa Gresova, Bulent Alten, Petr Volf, Vit Dvorak

**Affiliations:** 1 Laboratory of Entomology, Institute of Zoology, Chisinau, Republic of Moldova; 2 Department of Biology, Ecology Section, Faculty of Science, VERG Laboratories, Hacettepe University, Ankara, Turkey; 3 BioCeV, Institute of Microbiology of the Czech Academy of Sciences, Vestec, Czech Republic; 4 Department of Parasitology, Faculty of Science, Charles University, Prague, Czech Republic

**Keywords:** *Phlebotomus*, Bloodmeal analysis, Haplotype network, MALDI-TOF MS protein profiling

## Abstract

**Background:**

Phlebotomine sand flies (Diptera: Psychodiae) in the Republic of Moldova have been understudied for decades. Our study provides a first update on their occurrence, species composition and bloodmeal sources after 50 years.

**Methods:**

During 5 seasons (2013–2017), 58 localities from 20 regions were surveyed for presence of sand flies using CDC light traps and manual aspirators. Species identification was done by a combination of morphological and molecular approaches (DNA barcoding, MALDI-TOF MS protein profiling). In engorged females, host blood was identified by three molecular techniques (RFLP, *cytb* sequencing and MALDI-TOF peptide mass mapping). Population structure of most abundant species was studied by *cox*1 haplotyping; phylogenetic analyses of ITS2 and *cox*1 genetic markers were used to resolve relationships of other detected species.

**Results:**

In total, 793 sand flies were collected at 30 (51.7%) localities from 12 regions of Moldova. Three species were identified by an integrative morphological and molecular approach: *Phlebotomus papatasi*, *P. perfiliewi* and *Phlebotomus* sp. (*Adlerius*), the first being the most abundant and widespread, markedly anthropophilic based on bloodmeal analyses, occurring also indoors and showing low population structure with only five haplotypes of *cox*1 detected. Distinct morphological and molecular characters of *Phlebotomus* sp. (*Adlerius*) specimens suggest the presence of a yet undescribed species.

**Conclusions:**

Our study revealed the presence of stable sand fly populations of three species in Moldova that represent a biting nuisance as well as a potential threat of pathogen transmission and shall be further studied.

**Graphical abstract:**

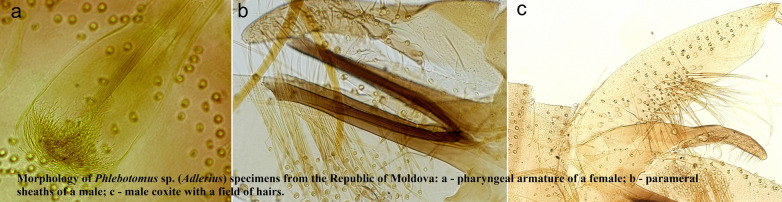

**Supplementary Information:**

The online version contains supplementary material available at 10.1186/s13071-021-04858-4.

## Background

Phlebotomine sand flies (Diptera: Psychodidae) are vectors of several pathogens including parasitic protozoans of the genus *Leishmania* and phleboviruses and thus of great importance in human and veterinary medicine [35]. In Europe, they are incriminated in *Leishmania* transmission, which occurs mostly in the Mediterranean countries where *L. infantum*, transmitted by several species of the subgenus *Larroussius*, is well established and where two other *Leishmania* species, *L. donovani* and *L. tropica*, have recently re-emerged [2]. However, potential expansion of sand flies due to climatic and environmental changes into regions where they were not previously established is expected in the near future [36], posing a risk of *Leishmania* introduction into non-endemic areas [47]. Recently, permanent sand fly populations were recorded in several countries north of their traditional distribution [38, 43], and the knowledge of sand fly fauna in long-time understudied regions like ex-Yugoslavia countries [14] and Romania [5] was significantly updated, further demonstrating the importance of entomological field research at the edge of their occurrence.

Moldova is considered a country non-endemic for leishmaniasis; there are no records of autochthonous human or canine cases [37]. In the past, presence of three *Phlebotomus* species was reported: *P. papatasi* (Scopoli), *P. perfiliewi* Parrot and *P. chinensis* Newstead [41, 42, 45]. However, the historical studies provided scarce information about their abundance and geographical distribution within the country, and there has been no update regarding species composition, spatial distribution and bloodmeal preferences of sand flies in Moldova since then. After several decades, increasing biting nuisance reported by residents from southern Moldova since 2011 initiated this study, which presents first data to our knowledge on sand flies in Moldova after 50 years.

## Methods

### Sand fly sampling

**Fig. 1:**
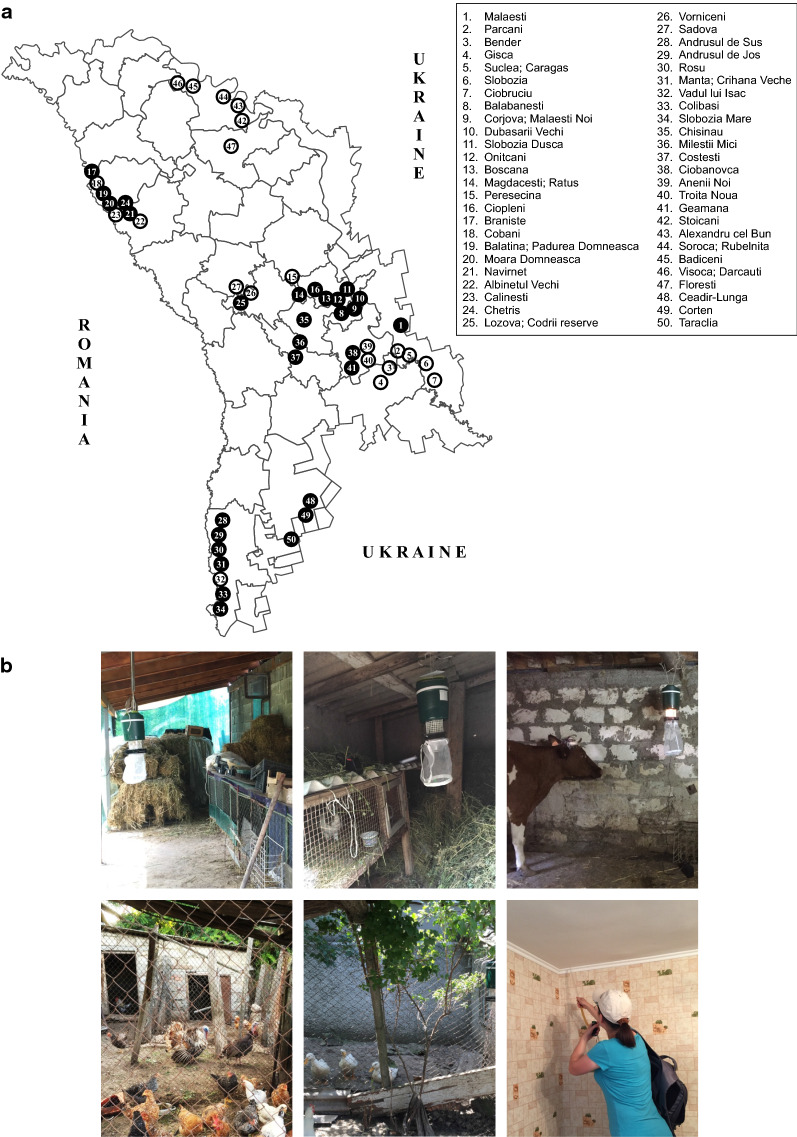
Sand fly collection localities and sites in the Republic of Moldova. **a** Map showing the sand fly collection locations. Settlements located close to each other marked under a same number (dots 5, 9, 14, 19, 25, 31, 44, 46 represent two localities). Black dots: positive sampling localities; white dots: negative sampling localities. **b** Sampling sites in rural localities of the study area

A countrywide field survey was conducted to assess the presence of sand flies at 58 localities from 20 regions between 2013 and 2017. Initially, field surveys were conducted at a rural locality in southern Moldova (July 2013, August 2014 and June–September 2015) and an urban area in central Moldova (July–September 2013) using miniature Centre for Disease Control (CDC) light traps (John W. Hock Company, model 512, Gainesville, Florida, USA) and manual aspirators (Additional file 1: Table S1). In 2015, two CDC light traps were operated on the same sites close to an animal shelter between June 22 and September 26, 2015. Cross-sectional entomological surveys were then conducted between 2016 and 2017. A total of 55 localities from 16 regions were surveyed in 2016 and 10 localities from 9 regions in 2017 (Fig. 1a). Sampling was performed using CDC light traps (Trappola per Monitoraggio Zanzare, IMT Original 2002, Italy) baited with CO_2_ (dry ice) and placed inside or outside of the animal shelters (livestock sheds, hen houses, dog kennels) (Fig. 1b). They were used overnight in the places protected from wind exposure. Entomological collections were complemented by mouth aspirators inside the houses and animal shelters. Mouth aspirators were also used within human dwellings and animal shelters. Each collecting site was numbered and mapped using a global positioning system (GPS). Ceadir-Lunga, a rural locality in southern Moldova, was selected for seasonal sand fly collections in 2015 and 2017 using CDC traps and manual aspirators (WGS84 coordinates: 46.06549 N, 28.84219 E). Two CDC light traps operated on two permanent sites in Ceadir-Lunga close to two poultry houses between June 22 and September 26, 2015. Each site was sampled 2–3 times per week; number of performed samplings was reduced to once per week in case of rain. Collection by manual aspirators inside houses (*n* = 13) and animal shelters (*n* = 14) was conducted several times per week between June 26 and September 14, 2015. Every time one house and/or one animal shelter was visited. In 2017, two CDC light traps operated every week on the permanent sites between July 6 and September 25. Additionally, sand flies were collected manually inside a chicken coop and a house (July 30–31, 2017).

### Morphological identification of sand flies

The collected insects were killed by freezing in dry ice and preserved in 70 or 96% molecular grade ethanol. For morphological identification, head and genitalia of each specimen were dissected and mounted on slides using CMCP-10 high viscosity mounting medium (Polysciences, Hirschberg, Germany) or Berlese mounting medium, and the rest of the body was stored in ethanol for molecular analyses. Species identification was based on decisive morphological characters using published keys and descriptions [32, 40]. For specimens belonging to the subgenus *Adlerius*, morphometric measurements of decisive characters on head and genitalia were done using a light microscope Olympus BX51 (Olympus Life Science, Waltham, USA) with a camera system Olympus D70. Morphological characters were measured using the QuickPHOTO MICRO 3.0 software (Promicra, Prague, Czech Republic) and compared with previously published values [3, 13].

### Molecular taxonomy of sand flies

The remaining body parts of sand flies were stored in 96% ethanol for DNA extraction that was done using High Pure PCR Template Preparation Kit (Roche Life Science, Penzberg, Germany). Species identity of selected sand fly specimens was further assessed by amplification of cytochrome oxidase I (*cox*1) of mtDNA region using LCO/HCO primer pair or amplification of the second internal transcribed spacer 2 (ITS2) using the primer pair JTS3/C1a following the protocols published by Folmer et al. [17] and Depaquit et al. [7], respectively. Relevant molecular markers were chosen for analyzed species depending on the availability of reference sequences, beside *cox*1, which is widely used as a universal DNA barcoding primer for sand flies [6]. ITS2 was used for *P. perfiliewi* as it was deployed in the previous study of *P. perfiliewi* complex [9]. PCR products were purified using a QIAquick PCR Purification Kit (Qiagen, Hilden, Germany) and directly sequenced in both directions using the same primers used for DNA amplification. Sequences were edited and aligned using the BioEdit (v.7.0.9) [22] alignment editor.

Statistical parsimony network analysis implemented in TCS method has been frequently used for revealing the genetic divergence at the intraspecific level. This method has also been proven as a robust and practical way for species delimitation in several taxa [23]. Using a nucleotide data set, this method calculates the maximum number of mutational steps to link the haplotypes in a parsimonious way with 95% probability. Thus, the DNA sequences of haplotypes belonging to the same species are grouped in a single network, while the DNA sequences of haplotypes representing different species are grouped in separate networks. *Cox*1 sequences obtained for *P. papatasi* were compared with those available in GenBank using BLAST algorithm to confirm species identification. Haplotype network for 36 *P. papatasi* specimens collected at 20 different localities (Additional file 2: Table S2) was constructed by PopArt [31, 30] using TCS method. The position of collected *P. perfiliewi* specimens within the species complex *Phlebotomus perfiliewi* (*s.l.*) was assessed by sequencing analysis of ITS2 for six specimens and comparison with sequences available in GenBank. *Cox*1 sequences obtained for *Phlebotomus* sp. (*Adlerius*) from Moldova and the sequences available for the other members of the subgenus in GenBank were analyzed together to construct a neighbor joining tree based on the Kimura's two parameter (K2P) substitution model in MEGA v.6.0 [46]. Relationship between the members of *Adlerius* subgenus was further evaluated by constructing parsimony networks using TCS method in PopArt.

### Identification of sand flies using MALDI-TOF mass spectrometry

Sample preparation and analysis by MALDI-TOF MS protein profiling followed a protocol optimized for sand flies [12]. In total, 20 specimens (10 collected in 2016, 10 collected in 2017) were analyzed. Thoraxes of dissected specimens were homogenized by a manual BioVortexer homogenizer (BioSpec, Bartlesville, USA) with sterile disposable pestles in 10 μl of 25% formic acid and briefly centrifuged at 10,000*g* for 15 s. Two microliters of the homogenate was mixed with 2 µl of freshly prepared MALDI matrix, which was an aqueous 60% acetonitrile/0.3% TFA solution of sinapinic acid (30 mg/ml, Bruker Daltonics, Bremen, Germany). One microliter of the mixture was then spotted on a steel MALDI plate in duplicate. Protein mass spectra were measured in a mass range of 4–25 kDa on an Ultraflex III MALDI-TOF spectrometer (Bruker Daltonics) as a sum of 2000 laser shots (20 × 100 shots from different positions of the sample spot) and analyzed by FlexAnalysis 3.4 software. For species identification and cluster analysis, the protein profiles were processed using MALDI Biotyper 3.1 and compared with reference spectra of an in-house database constructed using protein spectra of 25 different sand fly species. For MSP dendrogram creation, an individual main spectrum (MSP) was generated from each analyzed spectrum.

### Bloodmeal analysis

Bloodmeals of engorged females, all identified as *P. papatasi* and captured in 11 different villages mainly by manual aspirators, were analyzed by three molecular approaches, using two DNA-based techniques and a mass spectrometry approach. For morphological species identification, specimens were dissected and mounted on slides, and for molecular techniques, abdomens containing host blood were used. When morphological identification was not conclusive, species identification was further confirmed by MALDI-TOF MS protein profiling using remaining thoraxes of analyzed specimens. Additional file 3: Table S3 summarizes details about all engorged females analyzed for identification of blood origin, providing information about trapping methods, localization of the traps, potential hosts available at the trapping sites and methods applied to identify the bloodmeals.

Bloodmeals of engorged females collected in 2016, all identified as *P. papatasi*, were analyzed by combination of RFLP assay by HaeIII and HinfI restriction enzymes and sequencing of cytochrome B (*cyt b*). In total, 100 bloodfed females were analyzed, originating from 10 of the surveyed localities. These females were collected mostly indoors, inside either human dwellings (91 specimens) or hen houses (5 specimens); four specimens were collected by a trap placed outside an animal shelter. A 359-bp fragment of vertebrate *cyt b* gene was amplified using the modified vertebrate-universal specific primers cyt bb1/cyt bb2 [29, 34]. PCR amplification was performed as described by González et al. [19], using water instead of DNA template as a negative control to exclude contamination by the reagents. The PCR product was purified by QIAquick PCR Purification Kit (QIAGEN), and 15 µl was digested by 1 µl of the enzyme HaeIII or HinfI (New England Biolabs) at 37 °C for 20 min in 50 µl of total solution according to a protocol of the producer. Digested product was separated on 2% agarose gel and observed under UV light. For a proportion of randomly chosen specimens, the purified amplicons were sequenced in both directions using the same primers, cyt bb1/cyt bb2, to confirm the bloodmeal identification by RFLP.

Bloodmeals of 25 chosen engorged females collected in 2016 (8 specimens) and 2017 (17 specimens) at 4 localities (Ceadir Lunga, Corjova, Malaesti Noi and Slobozia Mare), all identified by morphology as *P. papatasi*, were analyzed by peptide mass mapping of host-specific hemoglobin peptides using MALDI-TOF mass spectrometry as described by Hlavackova et al. [24]. From dissected specimens, thoraxes were stored for confirmation of species identification by MALDI-TOF MS protein profiling, and abdomens were homogenized in 50 μl of distilled water (Merck KGaA, Darmstadt, Germany) by BioVortexer homogenizer (BioSpec); 10 μl of the homogenate was then incubated with 10 μl of 50 mM *N*-ethylmorpholine acetate buffer (pH 8.1; Sigma-Aldrich) and 100 ng of trypsin (Promega) at 37˚C for 30 min. After the digestion, 0.5 μl of the mixture was deposited on a MALDI plate in duplicate, air-dried and overlaid with 0.5 μl of MALDI matrix (aqueous 50% acetonitrile/0.1% TFA solution of α-cyano-4-hydroxycinnamic acid; 5 mg/ml; Bruker Daltonics). Peptide mass mapping spectra were acquired on an Ultraflex III MALDI-TOF instrument in the mass range of 700–4000 Da and calibrated externally using a peptide standard PepMix II (Bruker Daltonics). At least two peptides per sample were selected for MS/MS sequencing using LIFT technology. MS/MS data were searched against the SwissProt database subset of vertebrate proteins using in-house MASCOT 2.1 search engine (Matrix Science).

## Results

### Entomological survey

In total, 151 sites from 58 localities were sampled using CDC traps and manual aspirators, of which 65 (43.0%) sites were positive for sand flies (Additional file 1: Table S1). A total of 793 sand flies (34.2% males) were collected and identified between 2013 and 2017 from 30 (51.7%) localities belonged to 12 regions of Moldova (Fig. 1a).

Overall, 66.9% (534/793) of specimens were collected by manual aspirators from 31 sites belonging to 21 villages, inside the houses (381 specimens, 26 sites) and animal shelters (153 specimens, 5 sites) (Fig. 1b). Three sand fly species of the genus *Phlebotomus* were identified based on their morphological characters as described later: *P. papatasi*, *P. perfiliewi* and *Phlebotomus* sp. (*Adlerius*). The most abundant species was *P. papatasi* (*n* = 754/793; 265 males, 489 females), followed by *Phlebotomus* sp. (*Adlerius*) (*n* = 20; 3 males, 17 females) and *P. perfiliewi* (*n* = 19; 4 males, 15 females). *P. papatasi* was the most widely distributed species, present in 48.3% (*n* = 28) of all sampled localities and 41.7% (*n* = 65) of all collected sites from ten regions in Moldova. During the entomological surveys, the species was present in high numbers inside the hen houses and human dwellings located in central and southern Moldova. Overall, 71.9% (*n* = 542) of *P. papatasi* specimens were collected by manual aspirators inside the buildings. *Phlebotomus perfiliewi* was present in 13.8% (*n* = 8) of all sampled localities and 6.6% (*n* = 10) of all sites, located in six regions of the country. This species was sampled only by CDC light traps with or without dry ice, placed inside or outside the animal shelters with the potential animal hosts represented mainly by goats, poultry and dogs. *Phlebotomus* sp. (*Adlerius*) specimens were collected from 10.3% (*n* = 6) of all inspected localities belonged to four regions. This taxon was sampled mainly by CDC light traps; only two females were captured by manual aspirator inside a hen house and one male was collected inside the house.

A total of 225 and 47 sand flies were sampled in 2015 and 2017, respectively, in the locality Ceadir-Lunga. In total, 18 specimens of all three species were trapped using CDC traps between June 22 and September 26, 2015: *P. papatasi* (4 males, 5 females), *P. perfiliewi* (4 females) and *Phlebotomus* sp. (*Adlerius*) (5 females). First specimens were trapped in mid-July and last in mid-August. Collection by manual aspirators inside houses and animal shelters between June 26 and September 14, 2015, yielded 207 sand flies (140 specimens in the houses and 67 specimens in animal shelters). The highest number of sand flies (*n* = 160) of two species was collected in July in hen houses: *P. papatasi* (49 males, 108 females) and *Phlebotomus* sp. (*Adlerius*) (1 male, 2 females). Indoor sand fly activity was markedly longer, first specimens being collected at first aspiration in June 24 and last in September 14 which was the last day of collections by aspirators.

In 2017, a total of 18 sand flies were collected by two CDC traps, operated on the permanent sites between July 6 and September 25. The majority of specimens (*n* = 10) belonged to *P. papatasi* (3 males, 3 females), *Phlebotomus* sp. (*Adlerius*) (3 females) and *P. perfiliewi* (1 female) was collected in July. Seven sand fly specimens of two species [4 males of *P. papatasi* and 3 females of *Phlebotomus* sp. (*Adlerius*)] were sampled in August and one specimen (*P. papatasi* male) in September 2017. Manual collection of sand flies inside a chicken coop and a house (July 30–31, 2017) provided additional 29 *P. papatasi* specimens (12 males, 17 females). Numbers of specimens trapped by the CDC traps during the course of active sand fly season in 2015 and 2017, respectively, are shown on graphs in Additional file 6: Figure S2.

### Morphological species identification

**Fig. 2:**
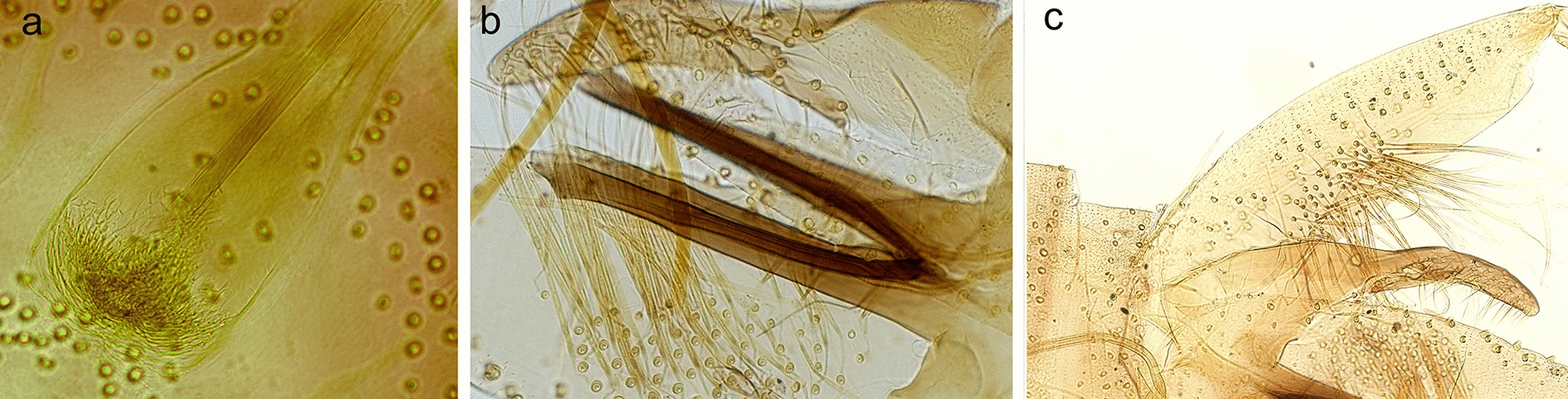
Morphology of *Phlebotomus* sp. (*Adlerius*) specimens from Moldova: **a** pharyngeal armature of a female (LCE6), **b** parameral sheaths of a male (LMG2), **c** male coxite with a field of hairs (LMG2)

Specimens of the subgenera *Phlebotomus* and *Larroussius* were all identified based on morphological characters on their head (pharyngeal armature) and genitalia (spermathecas of females, morphology of external terminalia of males) as *Phlebotomus papatasi* and *P. perfiliewi*, respectively. For *Phlebotomus* sp. (*Adlerius*) specimens, decisive morphological characters were compared with previously published values of described species within the subgenus, especially four species previously reported from Europe: *Phlebotomus balcanicus*, *P. creticus*, *P. longiductus* and *P. simici* (Table 1). Three analyzed males had markedly longer flagellomere 3 (A3) and labrum than the European species with *P. chinensis* and *P. longiductus* having the closest values of the remaining *Adlerius* species. Length of appendices on the external genitalia (coxite, style, parameral sheath) varied but did not match any of the *Adlerius* species completely; the number of coxite hairs was lower than for *P. balcanicus* and higher than for *P. simici* and *P. chinensis*. In 15 analyzed females, the length of A3 exceeded all that of *Adlerius* species except for *P. longiductus*, which also has a similar morphology of the pharyngeal armature (Fig. 2).

**Table 1:** Mean values obtained for the descriptive morphological characters for 3 males (A) and 15 females (B) of *Phlebotomus* sp. (*Adlerius*) in Moldova compared with other *Adlerius* species reviewed in Artemiev [3] and newly described *P. creticus* [13]

(A)	A3^a^	A3/Labrum^a^	Style length^a^	Coxite length^a^	Parameral sheath length^a^	Coxite /parameral sheath^a^	No. of coxite hairs
*Phlebotomus* sp. (*Adlerius*)	461.67	1.82	215. 95	436.75	183.67	2.38	60
	(439.44–493.20)	(1.69–1.86)	(207.10–223.00)	(402.10–461.95)	(176.47–191.60)	(2.20–2.61)	(57–64)
*P. balcanicus*	372.00	1.39	200.00	424.00	188.00	2.28	106
	(320.00–408.00)	(1.17–1.52)	(184.00–216.00)	(388.00–464.00)	(164.00–208.00)	(2.08–2.52)	(92–130)
*P. chinensis*	419.00	1.59	202.00	364.00	175.00	2.06	24
	(396.00–454.00)	(1.54–1.64)	(188.00–215.00)	(344.00–380.00)	(164.00–182.00)	(1.95–2.09)	(20–27)
*P. creticus*	340.00	1.29	x	405.20	195.30	x	69
	(285.00–406)	(1.14–1.53)	x	(345.00–451.00)	(178.00–219.00)	x	(54–85)
*P. longiductus*	417.00	1.52	204.00	386.00	195.00	1.98	64
	(340.00–500.00)	(1.31–1.83)	(180.00–236.00)	(340.00–428.00)	(172.00–240.00)	(1.64–2.23)	(51–81)
*P. simici*	275.00	1.18	148.00	296.00	189.00	1.57	20
	(240.00–290.00)	(1.10- 1.20)	(140.00–156.00)	(270.00–320.00)	(170.00–200.00)	(1.55–1.59)	(19- 22)

(B)	A3^a^	A3/Labrum^a^
*Phlebotomus* sp. (*Adlerius*)	397.80	1.26
	(269.90–458)	(0.98–1.52)
*P. balcanicus*	304.00	0.90
	(244.00–360.00)	(0.69–0.99)
*P. chinensis*	325.00	1.27
	(280.00–368.00)	(1.25–1.31)
*P. creticus*	298.00	0.88
	(268.00–326.00)	(0.80–0.94)
*P. longiductus*	388.00	1.06
	(332.00–440.00)	(0.93–1.30)
*P. simici*	244.00	0.80
	(200.00–270.00)	(0.75–0.86)

Minimum and maximum values of each measurement and count are given in parentheses

^a^Measurements in µm

### Species identification by molecular techniques

**Fig. 3:**
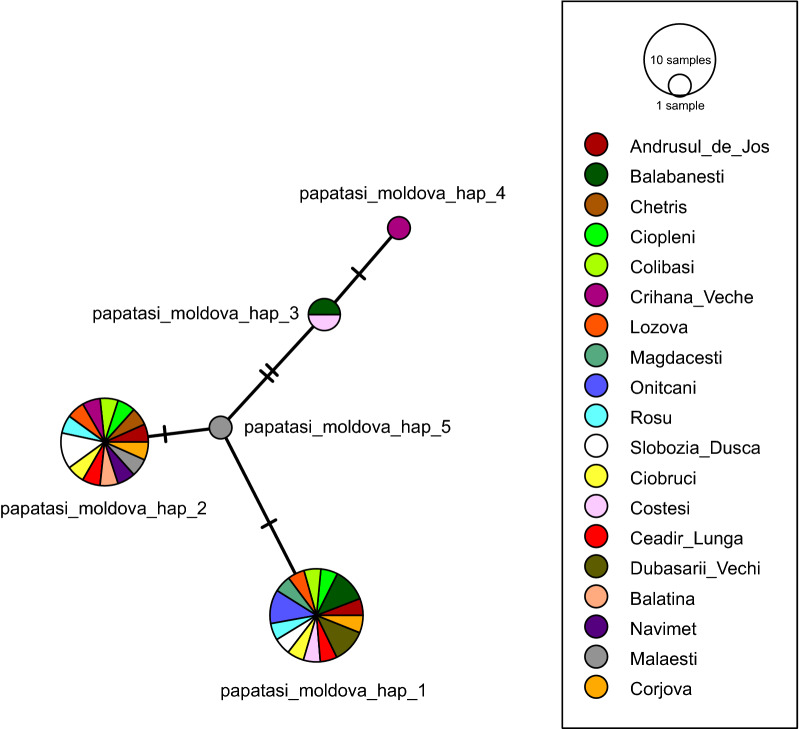
Haplotype network constructed for 36 *P*. *papatasi* specimens collected from different regions of Moldova. The relative frequency of haplotypes was reflected by the size of the circle, and dashes represent the mutational steps

DNA barcoding by *cox*1 of 36 randomly selected *P. papatasi* specimens provided an alignment 602 bp long, showing presence of five unique haplotypes at the sampling localities. Obtained sequences of all specimens morphologically identified as *P. papatasi* confirmed the species identification when compared with sequences from GenBank using BLAST algorithm (99.46%–100% identity). Statistical parsimony analysis placed five haplotypes into a single network. Two haplotypes (hap1 and hap2) are dominant and present at most of surveyed localities, while the other three (hap3, hap4 and hap5) have a restricted occurrence, two of them unique at a single locality, Malaesti and Crihana Veche, respectively. Among these five haplotypes, only five polymorphic positions were found (Fig. 3).

All six analyzed *P. perfiliewi* specimens provided identical sequences of ITS2, and a neighbor joining analysis with sequences of *P. perfiliewi* species complex available from GenBank placed them in a lineage of *P. perfiliewi* (*s.s.*) with populations from Greece and Ukraine.

**Fig. 4:**
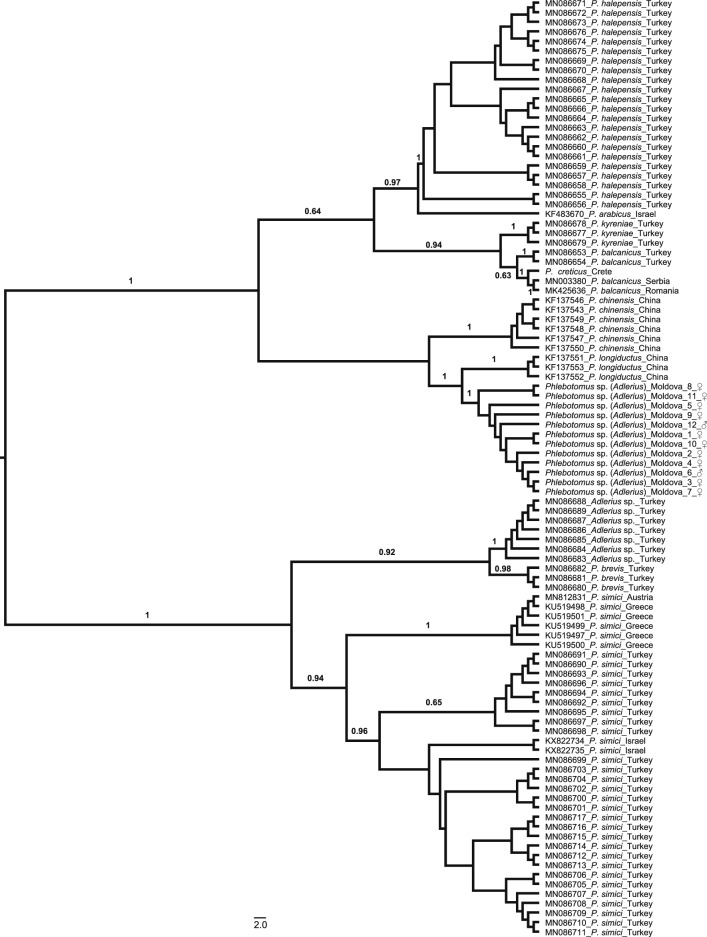
Neighbor joining dendrogram based on *cox*1 sequences showing relations among *Adlerius* species

**Fig. 5:**
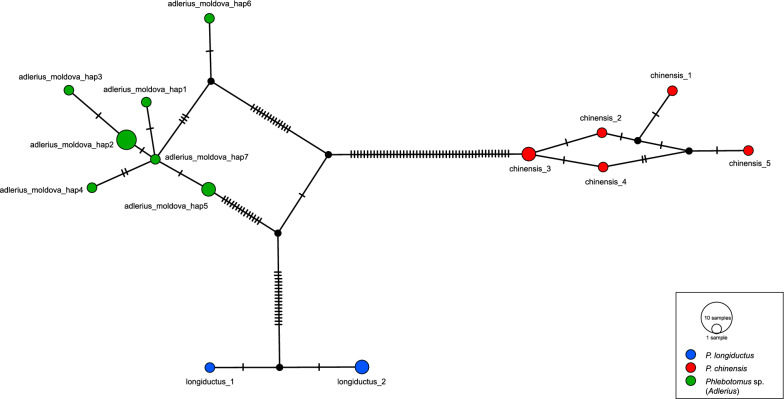
Haplotype networks constructed for 12 *Phlebotomus* sp. (*Adlerius*) specimens from Moldova, six *P. chinensis* specimens from China (GenBank Accession numbers: KF137543, KF137546–KF137550) and three *P. longiductus* specimens from China (GenBank Accession numbers: KF137551–KF137553). The relative frequency of haplotypes is reflected by the size of the circle; missing haplotypes are illustrated by the small circles, and dashes represent the mutational steps

The final alignment of the 12 *cox*1 sequences obtained for the *Phlebotomus* sp. (*Adlerius*) specimens collected from Moldova was 547 bp long. All the Moldovan *Phlebotomus* sp. (*Adlerius*) specimens were revealed to belong to the same species as they clustered together in the NJ tree and to have diverged from the rest of the *Adlerius* species included in the analysis (mean K2P distance: 6.5%–16.7%). The clade comprised *P. longiductus* from China, *Phlebotomus* sp. (*Adlerius*) from Moldova and *P. chinensis* from China, which was divided into three well-supported (100% bootstrap values) lineages (Fig. 4). Concordantly, the mean K2P distances between *Phlebotomus* sp. (*Adlerius*) from Moldova and *P. chinensis* and *P. longiductus* from China were higher (13.4% and 6.5%, respectively) than those yielded between some species pairs within the subgenus *Adlerius* (Additional file 4: Table S4). Supporting these results, TCS identified three independent networks for these three taxa. The first network included only the five *Phlebotomus* sp. (*Adlerius*) haplotypes from Moldova, while the second and third networks comprised *P. longiductus* (*n* = 2) and *P. chinensis* (*n* = 5) haplotypes from China, respectively. With a 95% connection limit, 32 mutational steps were required to connect Moldovan *Phlebotomus* sp. (*Adlerius*) to *P. longiductus* and 64 mutational steps to connect *Phlebotomus* sp. (*Adlerius*) from Moldova to *P. chinensis* (Fig. 5).

The NJ tree clearly showed that the recently identified *P. creticus* [13] is highly diverged from *Phlebotomus* sp. (*Adlerius*) from Moldova (mean K2P distance = 12.3%), clustering together with *P. balcanicus* sequences from Romania and Serbia. This group was placed as a sister taxon to *P. balcanicus* from Turkey (Fig. 4). The mean K2P distance between *P. creticus* and *P. balcanicus* from Romania and Serbia was lower (1.8%) than the one yielded between *P. creticus* and *P. balcanicus* from Turkey (4.5%) (Additional file 4: Table S4). These results were in concordance with the TCS results, which grouped *P. creticus* and *P. balcanicus* from Romania and Serbia in one network and constructed an independent network for *P. balcanicus* from Turkey (Additional file 5: Figure S1). *P. simici* specimens originated from Austria and Greece were clustered together, while Turkish and Israeli *P. simici* sequences were placed in a distinct lineage in the NJ tree, with high bootstrap support (> 95.0%). Rest of the taxa classified in the subgenus *Adlerius* were each represented by a single lineage (Fig. 4).

### MALDI-TOF mass spectrometric protein profiling

Species identification of chosen specimens from collections performed in 2016 and 2017 was confirmed by MALDI-TOF MS protein profiling. Of 20 analyzed, all except two produced intense, species-specific protein profiles of high quality.

Of material collected in 2016, protein profiles of four specimens originating from two localities (Balabanesti and Ciopleni) identified by morphology as *Phlebotomus papatasi* indeed matched with protein profiles of this species in the reference database with a log score value (LSV) > 2.0, which is accepted as the unambiguous assignment. Similarly, four specimens identified by morphology as *P. perfiliewi* (1 male and 1 female from Braniste, 2 females from Balabanesti) produced spectra similar to *P. perfiliewi* in the reference database with LSV > 2.0 and thus were conclusively identified as this species. Protein spectra of one male and one female of *Phlebotomus* sp. (*Adlerius*) originating from Magdacesti and Balabanesti, respectively, showed differences from spectra of all *Adlerius* species represented in the reference database (*P. arabicus*, *P. balcanicus*, *P. creticus*, *P. halepensis*, *P. simici*), indicating that Moldovan specimens represent another species within the subgenus *Adlerius*.

Of material collected in 2017, most effort was therefore focused on the further analysis of *Phlebotomus* sp. (*Adlerius*). Seven of ten analyzed specimens (1 male from Corten, 6 females from Ceadir-Lunga) produced identical protein profiles similar to those collected in 2016. In addition, protein profiles of one male of *P. papatasi* (locality Ceadir-Lunga) and two females of *P. perfiliewi* (locality Ceadir-Lunga and Slobozia Mare) were obtained, all similar with protein spectra of respective species obtained in the previous season.

**Fig. 6:**
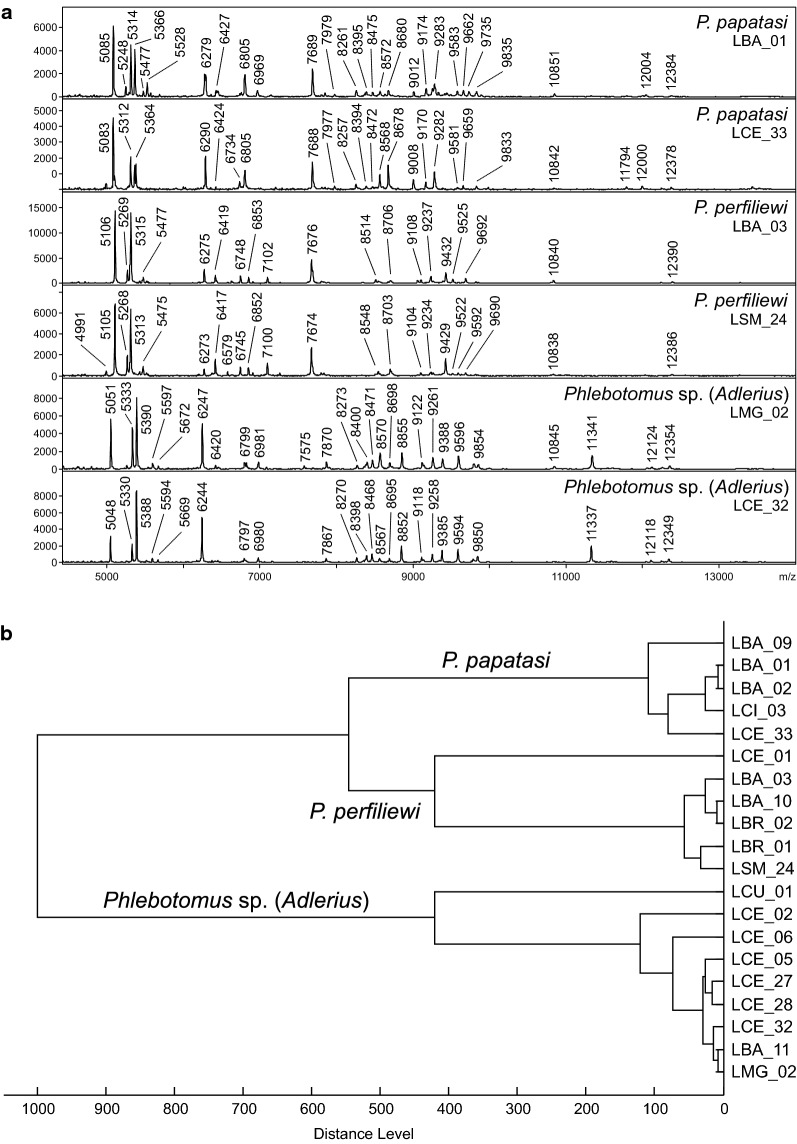
MALDI-TOF MS protein profiling of sand fly species in Moldova. **a** MALDI-TOF mass spectra of three typical Moldovan sand fly species. Zoomed mass range of 4–14 kDa is shown. **b** Cluster analysis of MALDI-TOF MS protein profiles of 20 sand fly specimens collected in Moldova. Distances in the dendrogram are displayed in relative units

**Fig. 7:**
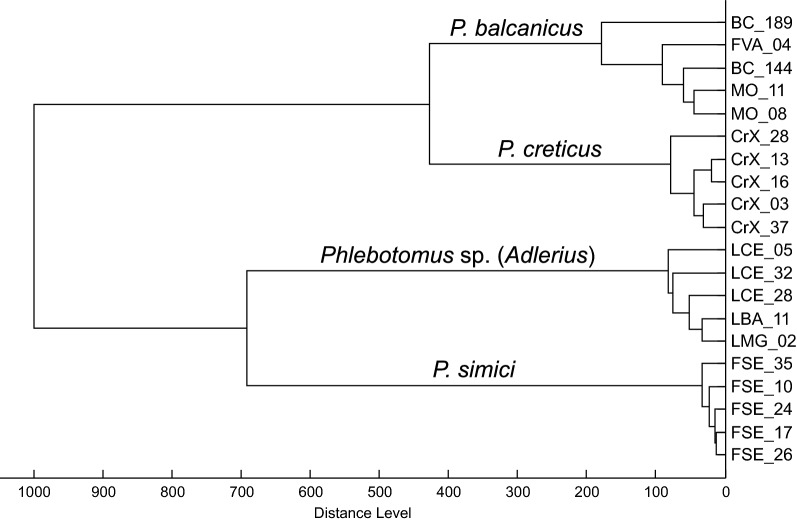
Dendrogram comparing MALDI-TOF mass spectra of *Phlebotomus* sp. (*Adlerius*) from Moldova and three other European members of the subgenus *Adlerius* (Bosnia and Herzegovina: specimen codes BC, Montenegro: specimen codes MO, Northern Macedonia: specimen codes FVA and FSE, Greece: specimen codes CrX). Distances are displayed in relative units

Specimens of three recorded species that belong to different subgenera of the genus *Phlebotomus* produced reproducible, distinctive and substantially different protein spectra (Fig. 6a), enabling rapid and conclusive species identification. No differences in the protein spectra of specimens from different localities were observed. The dendrogram constructed based on protein spectra of all 20 analyzed specimens shows clear clustering corresponding to these three species (Fig. 6b). It also demonstrates that two specimens with spectra of compromised quality [LCE01, a *P. perfiliewi* female from Ceadir-Lunga, and LCU01, a *Phlebotomus* sp. (*Adlerius*) male from Corten, both collected in 2017] were successfully and correctly identified, albeit positioned on long branches that reflect the lower quality of their spectra. Protein spectra of collected *Phlebotomus* sp. (*Adlerius*) specimens were compared with protein profiles of three species of the subgenus *Adlerius* recently known to occur in Europe: *Phlebotomus balcanicus* and *P. simici* from an entomological survey in Bosnia and Herzegovina, Montenegro and Northern Macedonia [14] and newly described *P. creticus* from Crete [13]. Figure 7 shows that specimens of all four species grouped in their own distinct clades, *Phlebotomus* sp. (*Adlerius*) forming a sister cluster with *P. simici* from Northern Macedonia.

### Bloodmeal analysis of engorged females

A total of 100 engorged *P. papatasi* females were captured in 10 villages in 2016. Analysis of bloodmeal sources by RFLP assay on a part of vertebrate *cyt b* gene provided human restriction patterns in 98 specimens: 91 females collected inside human dwellings by aspirators, 4 females trapped by a CO_2_-baited CDC trap outside an animal shelter with rabbits and poultry and 3 engorged females trapped inside hen houses with poultry. Two specimens collected inside a hen house provided a restriction pattern of chickens (*Gallus gallus*). Sequencing analysis of *cyt b* amplicons from 2 specimens tested as feeding on chickens and from 20 randomly chosen specimens originating from different localities confirmed all host identifications revealed by RFLP.

MALDI-TOF peptide mass mapping analysis of bloodfed females included specimens collected in 2016 (8 specimens previously analyzed by RFLP and *cyt b* gene sequencing as described above) and 2017 (17 specimens), providing distinct host peptide maps for 22 of 25 analyzed females. All successfully analyzed specimens displayed 8–16 signals of host-specific alpha- and beta-hemoglobin peptides, except one female with fewer host peptides, which nevertheless still enabled a conclusive identification of the blood source (Additional file 3: Table S3). The sequencing of the peptide fragments by tandem mass spectrometry (MS/MS) and subsequent database searching provided amino acid sequences of these host peptides and assigned the following blood origin: 6 chickens (*Gallus gallus*) in samples from Slobozia Mare, 2 humans from Corjova, 3 humans from Malaesti Noi, 3 humans and 8 chickens from Ceadir-Lunga. In samples collected in 2016, 7 of 8 analyzed specimens successfully provided host peptide maps, confirming all identifications of 5 human bloodmeals (2 from human dwellings and 3 from animal shelters) and 2 chicken bloodmeals (both from a hen house) as previously revealed by RFLP and *cyt b* gene sequencing. In samples collected in 2017, identifications were obtained for 15 of 17 analyzed specimens, showing 12 chicken bloodmeals (all from hen houses) and 3 human bloodmeals (1 from human dwelling, 2 from a hen house).

## Discussion

This study provides first data on sand fly species composition, spatial distribution and host preferences in Moldova after > 50 years, substantially updating reports that indicated the presence of three species (*P. papatasi*, *P. perfiliewi* and *P. chinensis*) without providing sufficient information about their geographical distribution [41, 42, 45]. Our study confirmed low sand fly species diversity reported in Moldova in the past, reporting presence of three species. For their species identification, an integrative approach was deployed that enables morphological and molecular identification in parallel, utilizing both DNA barcoding as a standard method of molecular typing and MALDI-TOF MS protein profiling, a rapid and cost-effective tool recently emerging as an alternative method for species identification of various organisms including medically important arthropods. It provided 100% correct species determination with most protein profiles of a high quality and stable for specimens collected at different localities and during two consecutive seasons, thus representing a sufficiently robust identification tool. Moreover, correct species determination of few specimens, which provided protein spectra of lower quality, was nevertheless also achieved. These results support the use of this combined taxonomical approach as suitable for processing sand flies collected through field entomological surveys.

Presence of two of three previously detected species in Moldova was confirmed by our sampling, *P. papatasi* being the most abundant and most widely distributed species. Scarce historical data mentioned low numbers of *P*. *papatasi* (5 females and 2 males) in Giurgiulesti village, Cahul region, southern Moldova, in 1946. In 1947, during the papataci fever outbreak in southern Ukraine (Reni region) and southern Moldova (Giurgiulesti), sand fly surveillance revealed the presence of low numbers of *P*. *papatasi* in other villages located in Cahul region (Chislita, Colibasi, Vadul lui Isac, Manta, Crihana Veche villages and Cahul town), but no exact numbers were given. Only one *P*. *papatasi* male was caught in Chisinau city in 1947 [45]. The Ukrainian sand fly control efforts between 1951 and 1954 focused on treatment of buildings in the villages affected by dichlorodiphenyltrichloroethane (DDT) and hexachlorocyclohexane, providing a rapid reduction of sand fly fever morbidity and significant decrease of sand fly densities [20]. Moreover, between 1948–1956, the national malaria eradication programs successfully used the same insecticides to combat the malaria vector *Anopheles maculipennis* (*s.l.*) in Moldova. It can be assumed that such widespread use of insecticides led to a decrease of the sand fly densities in Moldovan villages as well. Sand fly surveillance after the antimalaric campaign showed the presence of three sand fly species in four regions (Vulcanesti, Taraclia, Cahul and Ceadir-Lunga) in southern Moldova between 1957 and 1967. In total, 453 sand fly specimens were collected manually inside the buildings at 8 localities during that decade with average number of specimens per building between 0.7 and 5.7 [45]. Our manual collections inside the buildings in the same villages (Giurgiulesti, Slobozia Mare, Colibasi) or villages located close to the historical sampling sites show a higher average number of sand flies inside the buildings (Table 2).

**Table 2:** Comparison of historical sand fly collections in the Republic of Moldova (1957–1967) and recent collections (2015–2017)

Locality	Specimens collected manually (1957–1967)	Average no. of specimens per building (1957–1967)	Specimens collected manually (2015–2017)	Average no. of specimens per building (2015–2017)
Etulia	43	2.4		
Chismichioi	196	5.7		
Alexandru Ioan Cuza (Suvorovo, old name)	38	1.6		
Giurgiulesti	16	1.3	17^a^	5.6^a^
Cislita-Prut	18	1.2		
Valeni	8	0.7		
Slobozia Mare	108	4.2	49	16.3
Colibasi	Low numbers^b^	Low numbers^b^	32	16
Cahul city	Low numbers^b^	Low numbers^b^	23^c^	7.7^c^
Cairaclia	26	1.1		
Not specified	Low numbers^b^	Low numbers	243^d^	8.1^d^
Total	453		292	

^a^2019

^b^1947

^c^2016 in Rosu (neighboring village to Cahul city)

^d^2015–2017 in Ceadir-Lunga city

The presence of sand flies inside the human dwellings and animal shelters was first reported by residents from southern Moldova in 2011 when the local population started suffering from sand fly bites during the summer season. Our entomological surveys revealed the presence of *P. papatasi* in high numbers inside the human dwellings and hen houses in southern and central Moldova. Between 2013 and 2017, a total of 793 sand flies (34.2% males) were collected and identified from 30 (51.7%) out of 58 localities belonging to 12 regions of Moldova (Fig. 1a) by CDC traps and manual aspirators (Additional file 1: Table S1). The northernmost sites of *P. papatasi* collections were recorded at latitudes 47° 35ʹ–47° 47ʹ where the species was present in low numbers only in CDC trap collections; the northernmost record of *P. papatasi* sampling in the 1950s was in the Chisinau city at latitude 47°02' [45].

Comparison of historical records and our recent data shows a higher average number of sand flies inside the buildings, and the biting burden reported by the residents living at sand fly-infested sites indicates an increase of sand fly population in Moldova that probably successfully recovered after ceasing wide-scale applications of insecticides. Moreover, while breeding of livestock in the households was common in the countryside before the 1990s, deterioration of the economic situation led to a reduction of livestock, which was replaced by a large number of backyard poultry in the villages. Perhaps both socioeconomic and environmental changes promoted the increase of *P. papatasi* densities in the villages.

Continuous trapping in Ceadir-Lunga during the active sand fly season in years 2015 and 2017 showed markedly longer activity of *P. papatasi* indoors; specimens were collected from first aspirations in the second half of June until last collections in mid-September, suggesting that the actual indoor activity of *P. papatasi* may have been even longer. On the contrary, first specimens from outdoor trapping were collected in early July, despite earlier trapping efforts. In 2015, no specimens were trapped outdoors after mid-August, and in 2017, outdoor activity ceased by mid-September, despite later trapping efforts (Additional file 6: Figure S2). Low numbers of trapped specimens do not allow drawing robust conclusions regarding the seasonal dynamics, and few data for the species recorded in Moldova are available from other countries of a similar latitude; no information on seasonality was provided by a recent study in Romania [5], and only scant remarks on trapping dates were given by a sand fly survey in Hungary [16]. Recent study of seasonal dynamics of *P. mascittii*, a species so far not recorded in Moldova, at localities in Styria and Lower Austria of similar or higher latitude showed sand fly activity from early June until end of August, depending on the year and trapping locality [27]. Further effort that will include more localities is needed to better understand the seasonal dynamics of sand fly populations in Moldova.

We applied several methods to determine blood sources of collected bloodfed females of *P. papatasi*. Bloodmeal analysis of engorged *P. papatasi* females collected in a 2016 survey by RFLP assay demonstrated their strong anthropophily; 98% of successfully analyzed females were feeding on humans. This finding is not surprising as 91 of 98 females determined to feed on humans were collected by manual aspirators inside the houses. Interestingly, the remaining 7 females with detected human blood were sampled outside an animal shelter with rabbits and poultry (4 specimens) or even inside hen houses with poultry (3 specimens), suggesting that *P. papatasi* females preferred to feed on humans even when other hosts were available. Moreover, while domestic cats were present at two indoor sampling sites, they were not determined to be a source of blood. Domestic chickens were detected as a second and less frequently utilized host, chicken blood being identified in two females collected inside a hen house. These findings were confirmed by a sequencing analysis of chosen engorged specimens that included also females with alternative blood sources to humans available. Peptide mass mapping using MALDI-TOF mass spectrometry was recently introduced as a new and effective tool for bloodmeal identification of hematophagous arthropods [24]. Beside other advantages, this method in general provides very specific identifications in case of human bloodmeals within this study based on MS/MS sequencing of 3–4 unique peptides of human hemoglobins, thus excluding a misidentification induced by human skin debris contamination during the sample preparation, which may blur the results provided by DNA-based methods. With a vast majority of analyzed bloodmeals identified as of human origin, we decided to apply this MALDI-TOF mass spectrometry approach to re-analyze some samples collected in 2016 to exclude a possibility of contamination and analyze all engorged females collected in the following season. It proved to be well applicable, providing 22 identifications of 25 analyzed specimens, confirmed all identifications of human and chicken blood previously revealed by RFLP and *cyt b* gene sequencing among samples from 2016 and identified 12 chicken bloodmeals and 3 human bloodmeals among samples from 2017. In conclusion, bloodmeal analyses of engorged females confirmed both humans and chickens as hosts of bloodfeeding sand fly females, emphasizing the apparent tight trophic connection of *P. papatasi* with human dwellings. Our observations support the notion that this species is an opportunistic feeder with host preferences varying according to the availability and abundance of the blood sources but strongly anthropophilic when feeding close to or even inside human dwellings. A vast majority of females feeding on humans may be also partially explained by the absence of other domestic animals (sheep, goats, cattle) at catching sites in Moldova while these hosts were reported as additional blood sources to humans in previously studied foci of *P. papatasi* in other regions [25, 48]. *P. papatasi* is a species with markedly wide geographical distribution; however, studies based on several genetic markers showed that various geographical populations are relatively homogeneous and of limited genetic differentiation [8, 15]. More genetic variation was observed mainly among populations geographically separated by prominent natural barriers such as the High Atlas Mountains in Morocco [21]. In our study, *P. papatasi* collected at various sites in Moldova showed a limited genetic variability in cytochrome oxidase I gene, providing five haplotypes with only five polymorphic sites, suggesting a genetically homogeneous population. With respect to the geography of Moldova and adjacent regions that lack natural barriers of dispersal, we may expect a rather unrestricted gene flow with populations from neighboring countries. The very low mean intraspecific K2P distance (0.02%) obtained for *P. papatasi* specimens collected from Moldova was comparable with those previously reported from Greece, Cyprus [10] and Turkey (Erisoz Kasap et al. [26]) and further supports the conclusion that Moldovan *P. papatasi* comprise genetically close populations.

Two other recorded species, *P. perfiliewi* and *Phlebotomus* sp. (*Adlerius*), were present sporadically in CDC trap collections inside or outside the animal shelters. *Phlebotomus perfiliewi* is one of the main vectors of *Leishmania infantum* in the Mediterranean basin and in Central Asia [35]. It is regarded as a species complex of at least three species; however, recent taxonomic study failed to align their morphological and genetic characters, and sequencing analyses of two chosen markers (ITS2 and *cyt* B) provided incongruent phylogenies [9]. Unfortunately, *cox*1 was not used in that study; we therefore decided to deploy ITS2. Sequences obtained from specimens collected in Moldova suggest that they belong to *P. perfiliewi* (*s.s.*) and are closely related to populations from Greece and Crimea.

The subgenus *Adlerius* harbors over 20 described and formally undescribed species, females of which are usually considered indistinguishable morphologically, and their identification is often based on the identification of associated males [1, 3]. Previous historical record of *P. chinensis* in Moldova is questionable and shall be considered with respect to later evolvement of taxonomy within the subgenus. *Phlebotomus chinensis* is a species currently understood to occur only in China. In the past, however, this taxonomic designation was applied to sand fly populations from much broader geographical areas, which were later described as new *Adlerius* species [3]. Morphological analysis of males from Moldova, albeit limited to only three trapped specimens, suggests that they differ from *P. chinensis* as well as two of three European *Adlerius* species, *P. balcanicus* and *P. simici*, in several characters including number of coxite hairs. This was further confirmed by MALDI-TOF MS protein profiling, which added evidence that also a third European *Adlerius* species, only recently described *P. creticus*, provides species-specific protein profiles distinctively different from specimens from Moldova. In a dendrogram derived from their protein spectra (Fig. 7), *Phlebotomus* sp. (*Adlerius*) from Moldova clustered with *P. simici* while *P. creticus* formed a sister clade with *P. balcanicus*. For the *cox*1 sequences obtained for the *Phlebotomus* sp. (*Adlerius*) (both male and female specimens) from Moldova, the BLAST search revealed these sequences to be 93.78–94.15% identical to *P. longiductus* from China (GenBank accession numbers: KF137551–KF137553). The NJ analysis placed the Moldovan *Phlebotomus* sp. (*Adlerius*) and *P. longiductus* in highly diverged lineages (Fig. 4), and the mean K2P distance between these two lineages (6.5%) was comparable to those previously reported for different sand fly species classified in the subgenus *Adlerius* (Erisoz Kasap et al. [26]). These results are further corroborated by parsimony network analysis which identified two independent networks for these two lineages, suggesting they may represent two different species.

Supporting the sequencing data, both female and male *Phlebotomus* sp. (*Adlerius*) specimens were found to be different from *P. longiductus* morphologically as described above, although the statistical significance of this difference was not assessed. *P. longiductus* is widely distributed in China, Central and South Asia, and in the Middle East and has been previously reported from Eastern Europe (reviewed in [35]). Therefore, examination of representative specimens across its range is needed to reveal if these morphological and molecular differences reflect intraspecific geographical variation of local populations or the cryptic speciation within this taxon. The close relationship between *P. balcanicus* sequences from Eastern Europe (Romania and Serbia) and recently identified *P. creticus* needs further evaluation as well as the divergence of two lineages within the widely distributed *P. simici*, which has been recently recorded in Austria, suggesting possible northward spread in Europe [28]. An integrative approach deploying several molecular methods that study both DNA and proteins to complement the traditional morphological “golden standard” was recently successfully applied when describing *Phlebotomus creticus*, a novel species from the eastern Mediterranean [13], as well as studying sand fly fauna in East Africa [39]. Our findings further advocate its use as a valuable tool that provides better understanding of the taxonomy and biogeography of this morphologically challenging yet medically important sand fly subgenus.

Notably, five species of sand flies were recently reported from Romania [5] and six species are known from Ukraine (except Crimea) [3, 4, 42]. We may speculate that some of the species occurring in these countries may be detected by further entomological surveys also in Moldova, especially when wider surveillance is possible. While this study cannot provide robust data on the seasonality of three detected species, the results obtained in Ceadir-Lunga, a most comprehensively surveyed site located in the south of the country, suggest that sand fly activity indoors may start earlier than outdoors and last until mid-September. Following studies at different localities with sand fly presence will provide full understanding of seasonal sand fly dynamics in Moldova.

Moldova is so far regarded as a non-endemic country for leishmaniasis [37]. In the past, a single officially published imported human case was reported in a 1-year-old child who traveled with his parents from Rustavi, Georgia, to Straseni Region, Moldova, in the spring of 2013 [33]. In the 1980s, a total of 6000 samples of human serum from 28 regions in Moldova were tested for the presence of antibodies to sand fly fever viruses. In the Vulcanesti region (southern Moldova), 7.0% and 10.5% of humans tested were positive for antibodies to sandfly fever Sicilian virus (SFSV) and Neapolitan sandfly fever viruses, respectively [44]. Precise information on the imported/autochthonous human and canine cases of leishmaniasis in Moldova is not available. However, autochthonous cases of canine leishmaniasis have been recently reported in several countries in proximity to Moldova, which are also considered as non-endemic for leishmaniasis, namely Romania [11], Ukraine [18] and Hungary [47]. The Republic of Moldova continues to preserve the traditional rural lifestyle with domestic pets, poultry and cattle in backyard animal shelters, but with limited veterinary care. The presence of two proven vectors of leishmaniasis in the country and favorable environmental conditions may have an impact on the emergence and local transmission of leishmaniasis in Moldova. Intensive and continuous surveillance should be carried out to regularly update the information on sand fly vectors and the presence of *Leishmania* parasites in potential domestic and wild reservoirs and sand flies.

## Conclusions

Three sand fly species of the genus *Phlebotomus* were identified in the Republic of Moldova based on integrated morphological and molecular approaches. *Phlebotomus papatasi* was the most widely distributed and abundant species, feeding indoors and strongly anthropophilic as suggested on bloodmeal analyses. Haplotype network analysis showed low structuring of the *P. papatasi* population with only 5 haplotypes of *cox*1 with minor differences detected. Two other recorded species, *P. perfiliewi* and *Phlebotomus* sp. (*Adlerius*), were present sporadically in CDC trap collections. ITS2 sequences of collected *P. perfiliewi* specimens identified then as *P. perfiliewi* (*s.s.*). Distinct morphological and molecular characters of *Phlebotomus* sp. (*Adlerius*) specimens suggest the presence of yet undescribed species that will be further studied to reveal their relationships with other species within the subgenus and their potential role in pathogen transmission. The presence of two proven vectors of leishmaniasis in the country emphasizes a need for intensive and continuous surveillance to update the information on sand fly populations.

## Supplementary Information

**Additional file 1: Table S1.** Positive sand fly localities in the Republic of Moldova.

**Additional file 2: Table S2.** Information on *Phlebotomus papatasi* specimens used for haplotype network constructed by PopArt using TCS method.

**Additional file 3: Table S3.** Engorged *P. papatasi* females analyzed for identification of blood origin and indicates which methods were applied.

**Additional file 4: Table S4.** *cox*1 sequence divergence between *Phlebotomus* sp. (*Adlerius*) from Moldova and some members of the subgenus *Adlerius* based on K2P nucleotide substitution model. Intraspecific distances were indicated on the diagonal line.

**Additional file 5: Figure S1.** Haplotype networks constructed for *P. balcanicus* from Romania (GenBank Accession Number: MK425636), Serbia (GenBank Accession Number: MN003380), Turkey (GenBank Accession Numbers: MN086653- MN086654) and *P. creticus* from Crete (GenBank Accession Numbers: MT501623-MT501638). The relative frequency of haplotypes was reflected by the size of the circle; missing haplotypes were illustrated by the small circles, and dashes represent the mutational steps.

**Additional file 6: Figure S2.** Numbers of specimens trapped by the CDC traps during the course of active sand fly season in 2015 and 2017 in Ceadir-Lunga.

## Data Availability

All data generated or analyzed during this study are included in this article and its additional files, available as Additional file 1: Table S1, Additional file 2: Table S2, Additional file 3: Table S3, Additional file 4: Table S4, Additional file 5: Figure S1. *Cox*1 sequences obtained during this study were deposited in GenBank database under the accession numbers MZ519855-MZ519866.
