# Supplementary figures and images for: Phlebotomine sand fly survey in the Republic of Moldova: species composition, distribution and host preferences
Source: Parasit Vectors. 2021 Jul 21;14:371. doi: 10.1186/s13071-021-04858-4 (PMC8293551; doi:10.1186/s13071-021-04858-4)

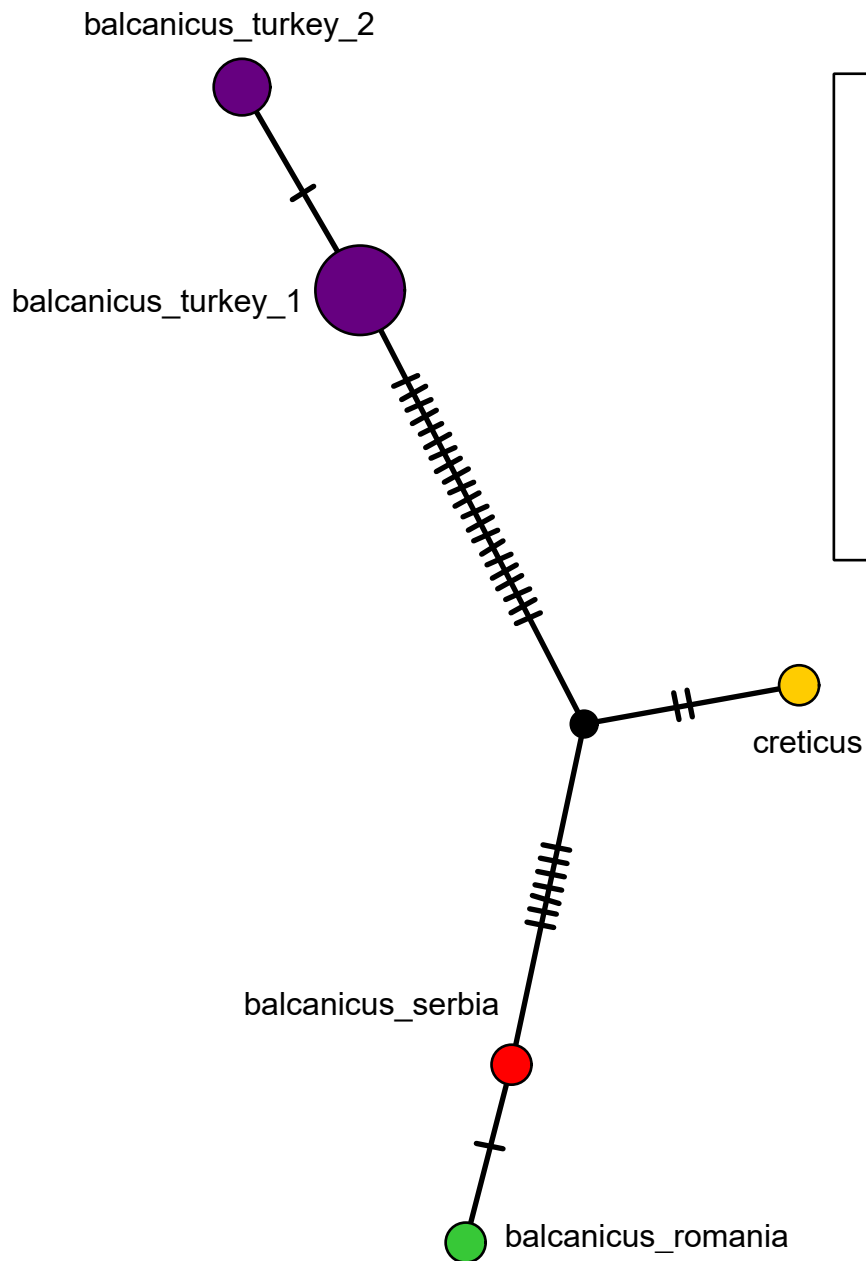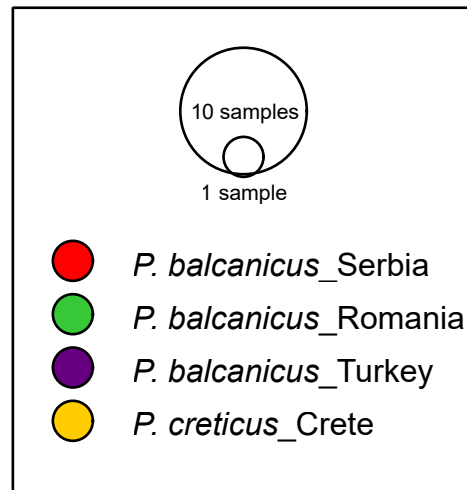

Supplement: Supplementary file 5 — Additional file 5: Figure S1. Haplotype networks constructed for P. balcanicus from Romania (GenBank Accession Number: MK425636), Serbia (GenBank Accession Number: MN003380), Turkey (GenBank Accession Numbers: MN086653- MN086654) and P. creticus from Crete (GenBank Accession Numbers: MT501623-MT501638). The relative frequency of haplotypes was reflected by the size of the circle; missing haplotypes were illustrated by the small circles, and dashes represent the mutational steps. [file 13071_2021_4858_MOESM5_ESM.pdf]
